# CD32 is enriched on CD4^dim^CD8^bright^ T cells

**DOI:** 10.1371/journal.pone.0239157

**Published:** 2020-09-22

**Authors:** Amber K. Virdi, Jennillee Wallace, Hannah Barbian, Maureen H. Richards, Ethan M. Ritz, Beverly Sha, Lena Al-Harthi

**Affiliations:** 1 Department of Microbial Pathogens and Immunity, Rush University Medical Center, Chicago, IL, United States of America; 2 Biostatistics and Bioinformatics Core, Rush University Medical Center, Chicago, IL, United States of America; 3 Infectious Diseases Division, Rush Medical College, Chicago, IL, United States of America; University of Texas Medical Branch at Galveston, UNITED STATES

## Abstract

CD4^dim^CD8^bright^ T cells, a genuine population of CD8^+^ T cells, are highly activated and cytolytic. Recently, the low affinity IgG Fc fragment receptor CD32a was described as marker of HIV latency while others reported that CD32a is associated with T cell activation. Given that we have previously established that CD4^dim^CD8^bright^ T cells are highly activated, mediate anti-HIV responses, and are infected by HIV, we assessed here CD32 expression on CD4^dim^CD8^bright^ T cells in context of HIV. CD32 frequency on peripheral CD4^dim^CD8^bright^ and CD4^+^ T cells was determined by flow cytometry among HIV negative and HIV positive patients. We report that among HIV^-^ individuals, mean CD32 percent expression was 60% on CD4^dim^CD8^bright^ T cells and 17% on CD4^+^ T cells (p<0.01). Among HIV^+^ patients, mean CD32 percent expression was 54% on CD4^dim^CD8^bright^ T cells and 12% on CD4^+^ T cells (p<0.001). CD32 expression on CD4^dim^CD8^bright^ T cells did not correlate with CD4 count and viral load and was not different by HIV serostatus. CD32 was also higher on other double positive T cell populations in both HIV negative and HIV positive donors in comparison to their single positive T cell counterpart. Together, these studies indicate that CD32 is enriched on double positive T cells regardless of HIV serostatus. The functional role of CD32 on these double positive T cells remains to be elucidated.

## Introduction

CD4 and CD8 expression on mature T cells is thought to be mutually exclusive. However, there is extensive body of literature demonstrating that mature CD8^+^ T cells, upon activation, upregulate CD4 de novo on their surface [1–13]. These cells have been termed CD4^dim^CD8^bright^ T cells because while the intensity of the CD8 molecule is similar to that of single positive CD8^+^ T cells, the CD4 molecule expression is lower than that of a single positive CD4 T cell. CD4^dim^CD8^bright^ T cells are not premature thymocytes as they do not express markers of immature T cells such as CD1a [10, 14]. The CD8 molecule on these cells is also αβ and not CD8αα, as reported in a double positive (CD4^+^CD8^+^) T cell population in the gut [15]. CD4^dim^CD8^bright^ T cells are highly activated [1]. In fact, activation of highly purified single positive CD8^+^ T cells to generate the CD4^dim^CD8^bright^ T cells phenotype is linked to induction of key markers of T cell activation, including HLA-DR, CD38, CD25, CD69, and Fas receptor CD95 [1]. Further, CD4^dim^CD8^bright^ T cells are increased during the aging process [16], in some autoimmune diseases [17], and in some viral infections [7]. We showed that CD4^dim^CD8^bright^ T cells are enriched among HIV infected individuals that naturally control HIV, known as long-term non-progressors (LTNPs) [8]. While CD4^dim^CD8^bright^ T cells makes up 3–5% of CD8^+^ T cells in healthy and chronically HIV infected individuals, among LTNPs they are elevated to 15% [8]. Most significantly this population is enriched in antiviral responses that are not necessarily specific for HIV, as CD4^dim^CD8^bright^ T cells constitute a significant population of anti-CMV and anti-HIV responses evaluated by MHC class I tetramer, polyfunctional responses, and surrogates for lytic activity (e.g. CD107α/β) [8]. Two additional characteristics may indicate that CD4^dim^CD8^bright^ T cells are a latent reservoir for HIV. 1) Due to their expression of CD4, they are infected by HIV [2] and 2) they robustly express β-catenin, a transcriptional co-regulator, demonstrated to inhibit HIV promoter activity [10, 18]. Together, these findings suggest that CD4^dim^CD8^bright^ T cells tether between anti-HIV immunity and potentially as a latent reservoir for HIV.

In this report, we evaluated the expression of CD32 on CD4^dim^CD8^bright^ T cells due to identification of CD32 as a putative marker of HIV latency. Albeit controversial, CD32 (FcγRII), is a family of low affinity IgG Fc fragment receptors commonly expressed on B cells, neutrophils, and monocytes [19] and contains three subsets of receptors, CD32a, b, and c. Due to its expression on antigen-presenting cells, activating receptor CD32a is thought to primarily function as a mediator of inflammatory immune responses such as cytolysis, phagocytosis, and degranulation [19]. While CD32 expression on T cells is well documented, its function on T cells is not fully defined [20]. Recently, CD32a expression on CD4^+^ T cells was proposed to be a marker of latently infected T cells, where in one study CD32^+^CD4^+^ T cells showed an enrichment in HIV DNA in therapeutically suppressed participants [21, 22]. Several groups since evaluated the efficacy of CD32(a) as a biomarker of HIV latency and were unable to demonstrate that CD32^+^CD4^+^ T cells were enriched in HIV DNA [23–26] or that CD32a acts as a biomarker of HIV latency. Rather, CD32 expression was more readily associated with activated CD4^+^ T cells [23, 24, 27], which is consistent with other studies explaining the expression of the CD32 Fc receptor family on T cells outside of the HIV field [28, 29].

In this report, we assessed whether CD32 is enriched on CD4^dim^CD8^bright^ T cells compared to CD4^+^ T cells between HIV negative and HIV positive groups. HIV seropositive donors included elite controller (undetectable viral loads for >5 years) or long term non-progressor (LTNP) (high viral load, CD4 > 500 cells/μL), combination antiretroviral therapy (cART) adherent (viral load <40 copies/mL), and unsuppressed viremic (cART non-adherent/treatment naïve) (viral load >10,000 copies/mL, CD4 count <500 cells/μL) participants. Our data demonstrates that CD32 expression is enriched on CD4^dim^CD8^bright^ T cells in comparison to CD4 single positive T cells independent of HIV serostatus.

## Materials and methodology

### Ethics statement

The studies involving human participants were reviewed and approved by Rush University medical center review board committee (IRBL06080703). The patients/participants provided written informed consent to participate in this study.

### Human peripheral blood mononuclear cells (PBMC) collection

Venous blood was collected from healthy controls and HIV positive patients visiting the Mark Weiss Memorial Clinic for Infectious Diseases at Rush University. Clinical measurements for CD4 were determined by flow cytometry, and plasma HIV-1 quantification was performed using the Abbot RealTime HIV-1 assay (lower limit of detection of 40 copies/mL) or the Quest HIV-1 Real-Time PCR assay (lower limit of detection of 20 copies/mL). Based on these counts and past clinical records, patients were defined as elite controllers (EC) (undetectable viral loads for >5 years), long term non-progressors (LTNP) (high viral load, CD4 > 500 cells/μL), combination antiretroviral therapy (cART) adherent (viral load <40 copies/mL), and cART non-adherent/ treatment naïve (viremic) (viral load >10,000 copies/mL, CD4 count <500 cells/μL). PBMCs were isolated using ficoll-hypaque density gradient centrifugation [11].

### Flow cytometry staining

PBMCs were first stained with LIVE/DEAD fixable aqua dead cell stain kit (Invitrogen, Carlsbad, CA). Cells were washed with DPBS and stained with fluorochrome conjugated antibodies from BD Biosciences (San Jose, CA)–CD3 (Pacific Blue, clone SP34-2), CD8 (APC-H7, clone SK1), CD32 (FITC, clone FL18.26)–and Biolegend (San Diego, CA)–CD4 (PE, clone OKT4). Samples were fixed in 1% PFA and run on a BD LSRFortessa (BD Biosciences, San Jose, Ca) and analyzed on FlowJo Software (TreeStar, Ashland, OR).

### Statistical analysis

Paired t-tests were applied to all data comparisons, with the exception of comparison of CD4^dim^CD8^bright^ and CD4 populations between HIV^+^ and HIV^-^ participants which was analyzed with an unpaired T-test. A Bonferroni correction was applied to account for multiple comparisons. ANOVAs were used for multiple comparisons across T cell populations. Tests were considered significant with p<0.05. All analyses were conducted in R version 3.6.0. All figures were constructed in GraphPad Prism (San Diego, CA).

## Results

### CD32 expression is higher on CD4^dim^CD8^bright^ T cells in comparison to CD4 single positive T cells, regardless of HIV serostatus

**Fig 1:**
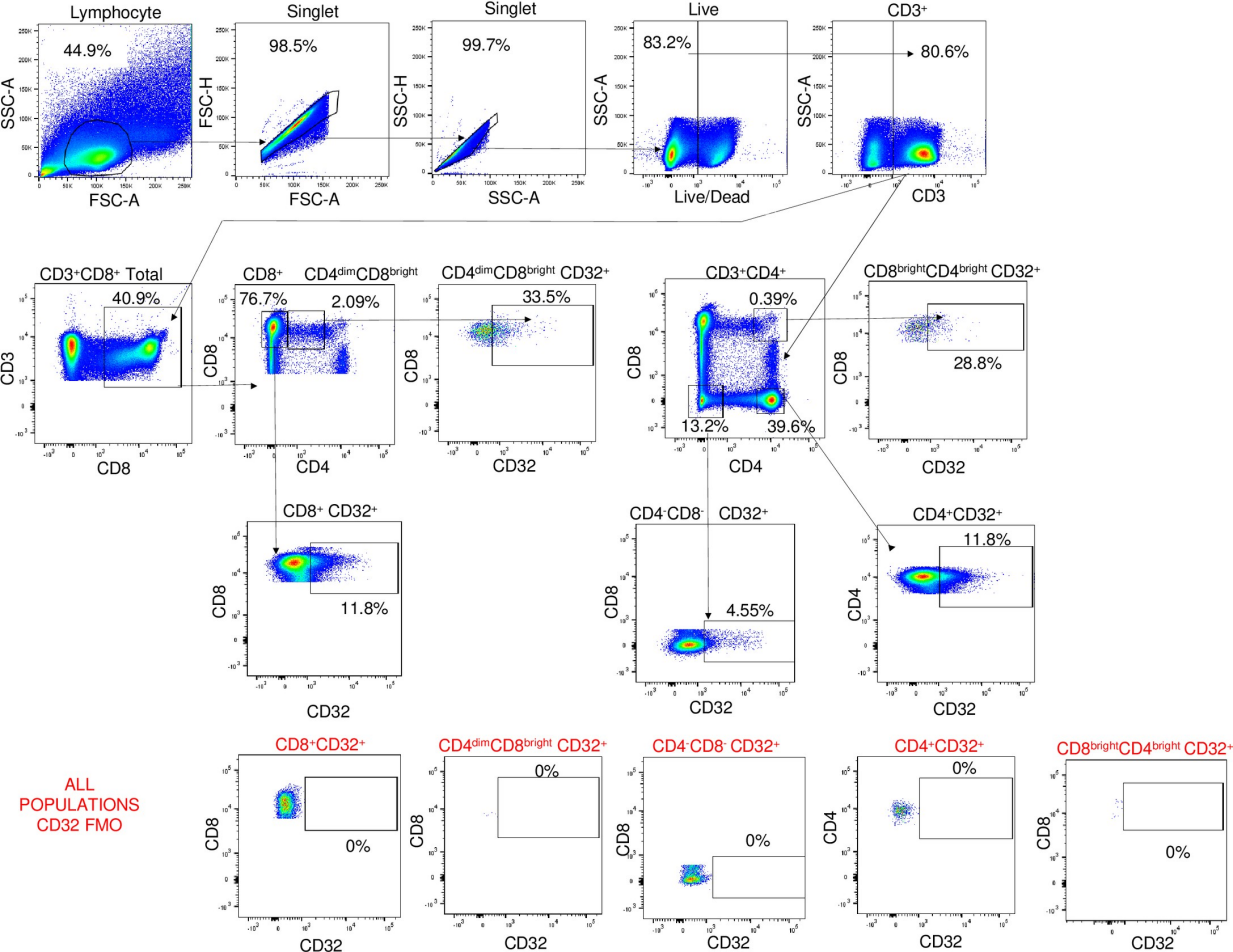
Gating strategy for CD32 expression on peripheral CD4^+^ and CD4^dim^CD8^bright^ T cell populations. Lymphocytes were gated from whole human PBMCs using forward and side scatter area, then two singlet gates were utilized to remove doublet populations. This was followed by an Aqua Live/Dead stain and total CD3^+^ cell gate. From this, two different gates were determined: total CD3^+^CD8^+^ cells and CD3^+^ CD4 single positive cells. From the CD3^+^CD8^+^cells, CD8 single positive T cell and CD4^dim^CD8^bright^ T cell populations were obtained. CD4 single positive T cells, CD4brightCD8bright, and CD8^-^CD4^-^ were gated from total CD3^+^ cells. The results of these gates are five T cell populations: CD8^+^, CD4^dim^CD8^bright^, CD4^+^, CD4^bright^CD8^bright^, and CD8^-^CD4^-^. CD32 expression was determined through usage of a CD32 FMO, which was used as a negative control for CD32 staining and allowed for accurate gating on the positive populations only.

**Fig 2:**
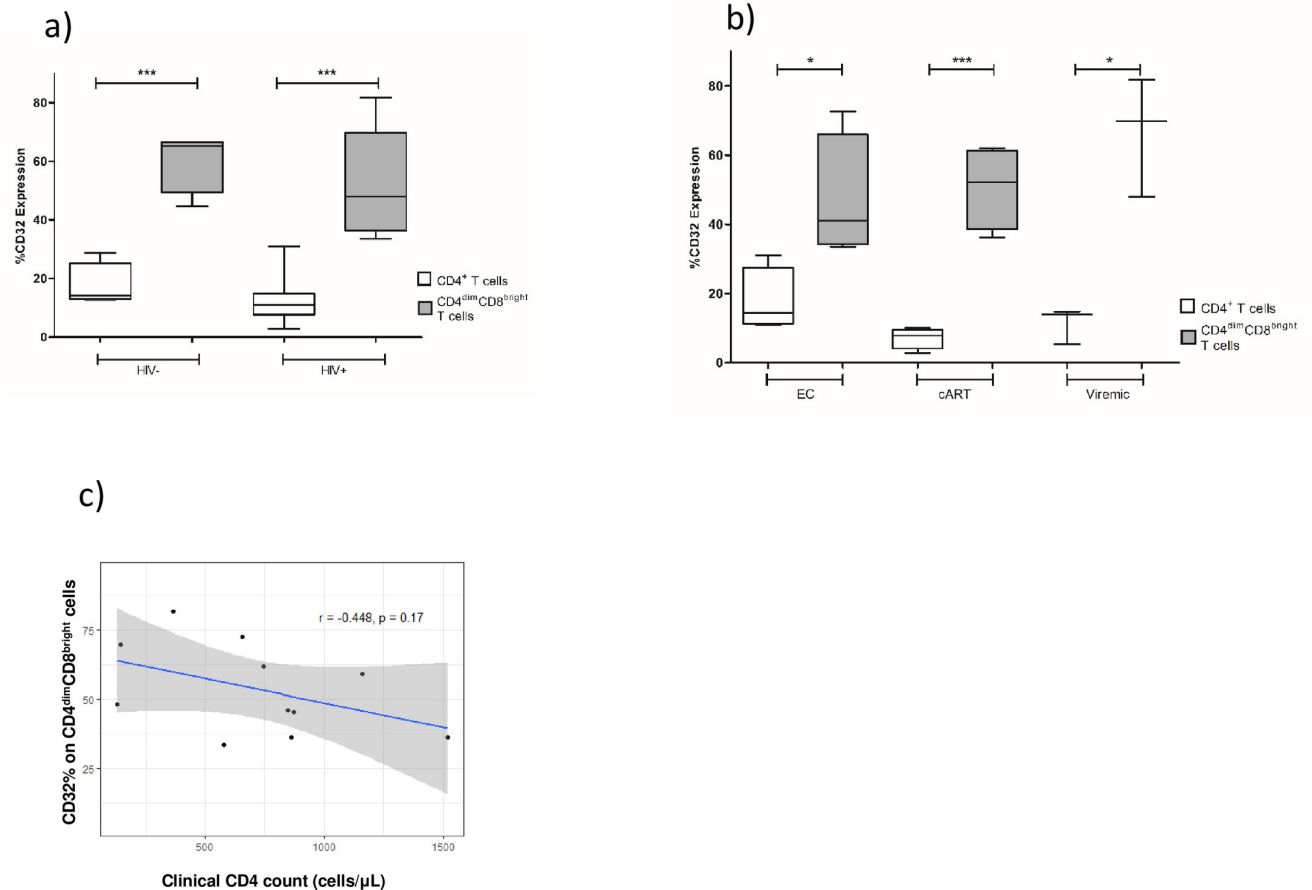
CD32 expression on CD4^+^ and CD4^dim^CD8^bright^ T cells among HIV seronegative and seropositive donors. **a**) CD32 expression on CD4^dim^CD8^bright^ T cells and CD4^+^ T cells from HIV^+^ and HIV^-^ blood donors was evaluated by flow cytometry using peripheral blood of HIV seronegative (n = 4) and HIV seropositive (n = 11 donors). **b**) PBMCs from elite controllers (EC) (undetectable viral loads for >5 years) including one long term non-progressor (high viral load, CD4 > 500 cells/μL), cART adherent (viral load <40 copies/mL), and cART non-adherent/ treatment naïve (viremic) (viral load >10,000 copies/mL, CD4 count <500 cells/μL) donors were evaluated by flow cytometry for CD32 expression. **c**) Relationship between CD32 expression on CD4^dim^CD8^bright^ T cells and CD4 count of HIV^+^ participants. Clinical CD4 counts taken on day of blood draw. * denotes p<0.05 determined by unpaired T-test and *** denotes p<0.01 as measured by paired T-test.

To assess the frequency of CD32 on CD4^dim^CD8^bright^ T cells and CD4^+^ single positive T cells (CD4^+^ T cells) in context of HIV serostatus, we isolated PBMCs from HIV seronegative donors (n = 4) and HIV^+^ donors (n = 11) and expression of CD32 on these populations was evaluated by multicolor flow cytometry. The CD4 counts and viral loads of HIV^+^ participants are shown in Table 1. The CD4 count ranged from 129–1,518 cells/μL and HIV viral RNA loads from undetectable to moderate (Table 1). Within the HIV^+^ group, there are three distinct subgroups of HIV positive donors: Elite controllers (including 1 long-term non progressor) (n = 4), cART suppressed (n = 4), and viremic, non-suppressed (n = 3) (Table 1). The gating strategy to delineate the CD4^dim^CD8^bright^ T cells and other CD4^+^ T cells subsets is depicted in Fig 1. Briefly, whole PBMCs were first sorted for lymphocytes based on forwards and side scatter, then single cells were gated for twice so that there were no confounding doublet T cells. Live, CD3^+^ T cells were then gated from singlets. From CD3^+^ cells, total CD8^+^ cells were gated out and from those CD8^+^ and CD4^dim^CD8^bright^ T cells were determined. Three other T cell populations were gated from CD3^+^ cells, including CD4^+^, CD4^bright^CD8^bright^, and CD4^-^CD8^-^ cells. For each of these T cell populations, CD32 expression was determined using a CD32 fluorescence minus one (FMO) as a negative control for CD32. Our analyses focused on populations that express CD4 because it’s a receptor for HIV. Among HIV^-^ individuals, mean percent CD32 expression was significantly higher on CD4^dim^CD8^bright^ T cells compared to CD4^+^ T cells (60.45% vs. 17.45%, respectively, p = 0.004) (Fig 2A). Similarly, among HIV^+^ individuals, mean percent CD32 expression was significantly higher on CD4^dim^CD8^bright^ T cells than CD4^+^ T cells (53.69% vs. 12.11%, respectively, p = <0.0001) (Fig 2A). Although the n was small, when the data was analyzed in relation to clinical status, mean percent CD32 expression on CD4^dim^CD8^bright^ T cells was still significantly higher among all the clinical categories of HIV seropositive donors in comparison to CD4^+^ T cells (EC: 47.08% vs. 17.68% (p<0.05), cART: 50.68% vs. 7.12% (p<0.005), and viremic: 66.53% vs. 11.35% (p<0.05). CD32 on CD4^dim^CD8^bright^ T cells was not significantly different between the various HIV clinical statuses (p>0.05) (Fig 2B). Given the low number of donors in each category, this finding does not exclude that with a higher n a relationship may emerge. Elite controllers, however, are a rare population of people living with HIV and are challenging to capture. At Rush’s infectious disease clinic, there is a total of 10–15 elite controllers, constituting approximately 1% of HIV^+^ patients seen at the clinic and not all consent to participate in any given study. These data demonstrate that CD32 expression is enriched on CD4^dim^CD8^bright^ T cells in comparison to CD4^+^ T cells in both HIV negative and HIV positive individuals.

**Table 1:** Clinical description of study participants.

Subject ID	HIV Disease Status	CD4 Count (cells/μL)	HIV RNA (cp/mL)
1	Negative	n/d	n/d
2	Negative	n/d	n/d
3	Negative	n/d	n/d
4	Negative	n/d	n/d
4–2	Elite Controller	849	<20
7–3	Elite Controller	655	<20
8	Long Term Non-Progressor	1,518	6,857
28	Elite Controller	578	<40
11	cART Suppressed	873	<40
15	cART Suppressed	745	<40
19	cART Suppressed	1,161	<20
21	cART Suppressed	862	<40
14	Viremic, Uncontrolled	146	30,274
20	Viremic, Uncontrolled	129	21,962
25	Viremic, Uncontrolled	365	14,158

HIV^-^ (n = 4) and HIV^+^ subjects (n = 11) were included in the study. HIV^+^ participants were categorized based on clinical markers of CD4 count and HIV RNA load. n/d = not determined.

### CD32 expression on CD4^dim^CD8^bright^ T cells is not associated with CD4 count or viral load among HIV^+^ patients

We next evaluated the relationship between CD32^+^ CD4^dim^CD8^bright^ T cells, clinical CD4 count, and viral load among HIV^+^ participants. Clinical markers were determined on the day of blood draw. We found no correlation between CD4 count and CD32 expression on CD4^dim^CD8^bright^ T cells (r = -0.45, p = 0.17, Fig 2C). We also found no relationship between CD32 expression on CD4^dim^CD8^bright^ T cells and viral RNA load among participants with detectable viremia (participant 8, 14–25, Table 1) (p = 0.6, data not shown). These data indicate that enrichment of CD32 on CD4^dim^CD8^bright^ T cells is not associated with CD4 count nor HIV load.

### CD32 expression is higher on various populations of double positive T cells than single positive CD4 or CD8 T cells

**Fig 3:**
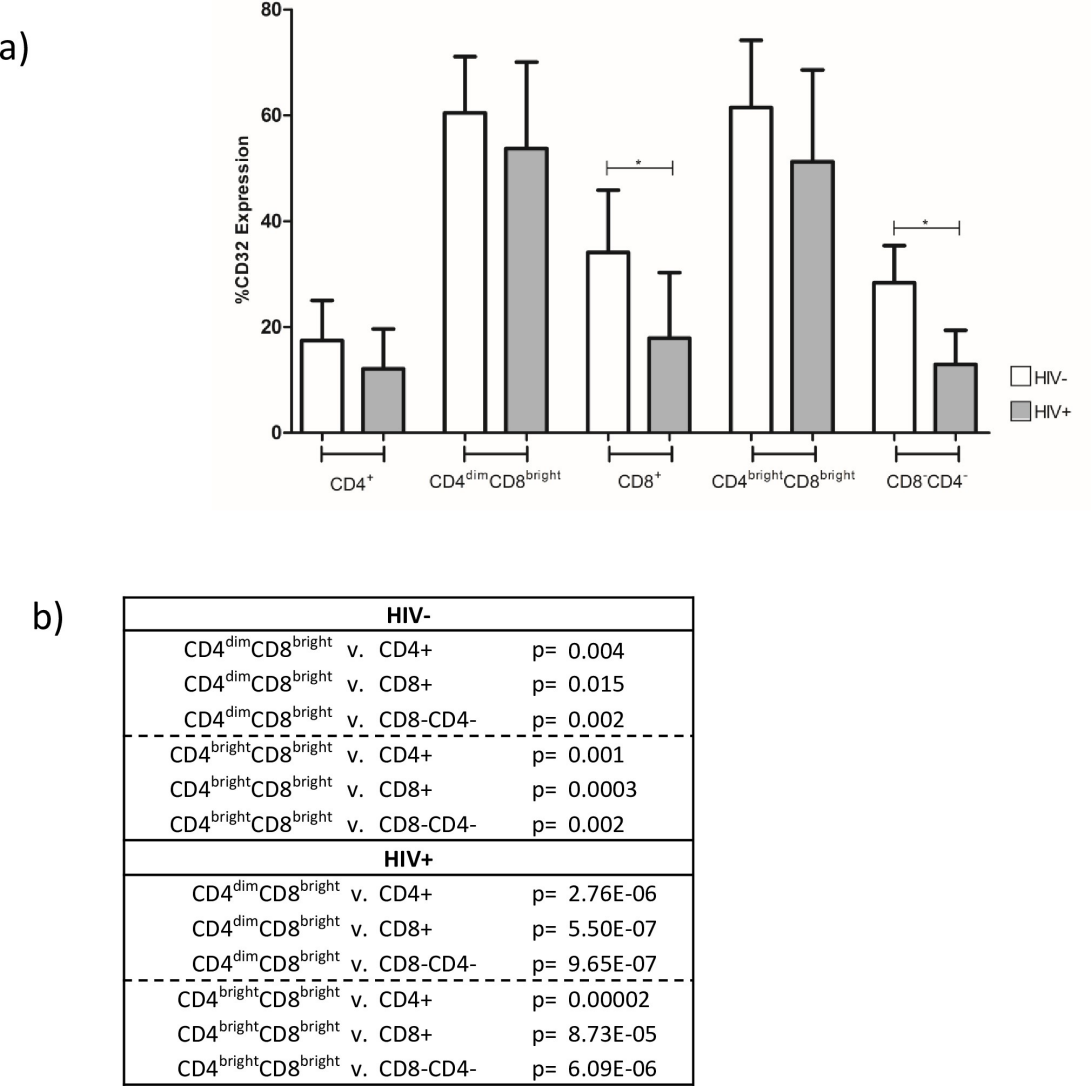
CD32 expression is higher on various CD4 and CD8 double positive T cell subsets. a) CD32 expression was determined on five T cell populations: CD8^+^, CD4^dim^CD8^bright^, CD4^+^, CD4^bright^CD8^bright^, and CD8^-^CD4^-^ cells. Positive CD32 was gated using FMO as a negative control. Expression of CD32 on each population was compared between HIV^-^ and HIV^+^ donors. * denotes p<0.05 as measured by unpaired T-test. b) Within group comparisons of CD32 expression on T cell populations in HIV^-^ and HIV^+^ donors was determined via ANOVA.

Although CD4^dim^CD8^bright^ T cells is the population shown to exert anti-HIV immunity and be infected by HIV, there are multiple CD4 and CD8 double positive T cells with varying intensity of CD4 or CD8 (Fig 1) [30]. We evaluated CD32 expression on these various subsets among HIV negative and HIV positive donors, as well as CD8^-^CD4^-^ T cell population and single positive CD4 and CD8 T cells. CD32 expression on CD8^+^ and CD8^-^CD4^-^ cells was lower in HIV^+^ individuals compared to HIV (Fig 3). In both HIV negative and HIV positive individuals, expression of CD32 on CD4^dim^CD8^bright^ and CD4^bright^CD8^bright^ T cells was higher compared to CD4^+^, CD8^+^, and CD8^-^CD4^-^ cells (Fig 3B). These studies indicate that T cell populations co-expressing CD4 and CD8 are enriched for CD32 compared to cells that are single positive or do not express either marker.

## Discussion

CD4^dim^CD8^bright^ T cells are a rare population in blood. This population exerts potent anti-HIV immune responses yet it is infected by HIV [2, 4, 5, 31, 32]. The infection may be latent in CD4^dim^CD8^bright^ T cells because they express β-catenin which induces de novo expression of CD4 on CD8^+^ T cells [33] on hand and inhibits HIV at the transcriptional level at the other [10]. Specifically, we previously showed that β-catenin forms a multiprotein complex on the HIV LTR with TCF-4 and SMAR and inhibits Pol II docking on the HIV promoter [10]. As such. CD4^dim^CD8^bright^ T cells may be a candidate latent reservoir for HIV. Because they are rare and cannot be sorted directly as they down regulate CD4 expression on their surface in response to cell sorting, we assessed whether they may be enriched in CD32, a potential biomarker of HIV latently infected cells.

To date, there is no consistent marker that can delineate HIV latently infected cells. Such a marker is highly valuable to assess the size of the HIV reservoir, at least in T cells, as there are significant sanctuary sites in non-lymphoid tissues that exist for HIV [34, 35]. A biomarker of latency is also valuable for HIV cure strategies. CD32 remains a controversial markers of HIV latency. Since the publication of the Descours et al. study reporting that CD32a is a biomarker of HIV latency in T cells, several groups have not been able to confirm this claim [23, 24, 26, 27, 36–38]. Of note, Abdel-Mohsen et al. determined that CD32 expression on CD4^+^ T cells is enriched on a subset of transcriptionally active CD4^+^ T cells and that the CD32^+^CD4^+^ T cell population contributed significantly less to the total pool of HIV DNA within the CD4^+^ T cell population [23]. Badia et al. found that the expression of CD32 on CD4^+^ T cells is not specific to HIV infection and CD32 expression was associated with T cell activation in the context of HIV infection [24]. Another study, Martin et al., assessed peripheral CD32^+^ CD4 T cells and found enrichment of activation marker HLA-DR and HIV co-receptors CCR5 and CXCR4 in both HIV^+^ and HIV^-^ samples [36]. A recent study, however, supported the original finding of Descours et al. and most importantly demonstrated that optimization of CD32^+^CD4^+^ T cell isolation, utilizing multiple rounds of first negative CD4 selection followed by CD32 positive selection, is critical to reveal HIV DNA enrichment among CD4^+^ T cells [22]. They further indicated that the stringent sorting approach of CD4CD32^+^ cells is necessary to eliminate any CD32b^+^ B cells that may impact provirus quantification.

Although our focus is on CD4^dim^CD8^bright^ T cells, a CD4^bright^CD8^bright^ population is found in the blood. The anti-HIV immune responses, however, are within the CD4^dim^CD8^bright^ T cell population [8, 30]. CD32 was also enriched in the CD4^bright^CD8^bright^ T cell population in comparison to either single positive CD4 or CD8^+^ T cells, again regardless of HIV serostatus. CD32a is primarily expressed on innate immune cells such as neutrophils, monocytes, and macrophages [19, 39]. It is an activating receptor, known to mediate functions such as degranulation, phagocytosis, and cytolysis and at least for CD4^+^ T cells it may play a role in CD4^+^ T cell effector function and activation [20, 28, 29]. Its higher expression on these two double positive populations (CD4^dim^CD8^bright^ and CD4^bright^ CD8^bright^) is not clear, especially since it was higher on these populations that their single positive CD4 or CD8^+^ T cells. It may be an indicator of activated T cells or another specialized function.

Further, because CD32 expression was not different on these cells between HIV seropositive and sero negative donors, this suggest that it not be a biomarker of HIV latency or that HIV it may preferentially target CD32^+^ CD4 expressing T cells, no matter what the intensity of CD4 on these cells is. Definitive studies require isolation of these CD4^dim^CD8^bright^ T cells from HIV positive individuals, although it is challenging as they down regulate CD4 post sorting and their frequency is not high among chronically infected individuals. Activation markers have specific functions. For example, CD38 not only regulates cell activation, but also cell migration and has been associated with poor prognosis in diseases such as HIV [40, 41]. HLA-DR is induced on activated cells to heighten antigen presentation. Here CD32 is likely conferring an unknown function that facilitates adaptive immunity and it may be highly specific to the role of double positive T cell subsets in immunity.

Lastly, there are few caveats to our study. the expression of CD32 that we noted on the various T cell populations is higher than was previously reported [22] and this may be because we used a different clone of CD32 antibody. Both antibody clones are pan-reactive and neither can specifically define CD32a as there are currently no CD32a specific antibodies for flow cytometric analyses. We also did not find a difference in CD32 expression among HIV^+^ donors with various clinical categories of HIV (EC, virmeic, on cART) and this may because breaking the HIV^+^ cohort (n = 11) into various stages reduced the number within each category. A larger cohort may be able to decipher if any differences exist. Nonetheless, the finding that CD32^+^ CD4^dim^CD8^bright^ T cells is not different between HIV negative and HIV positive patients suggest that there may be a role of CD32 on these cells that is independent of whether it is a biomarker of HIV latency.
